# Biological Effect of Licochalcone C on the Regulation of PI3K/Akt/eNOS and NF-κB/iNOS/NO Signaling Pathways in H9c2 Cells in Response to LPS Stimulation

**DOI:** 10.3390/ijms18040690

**Published:** 2017-03-23

**Authors:** Sara Franceschelli, Mirko Pesce, Alessio Ferrone, Daniela Maria Pia Gatta, Antonia Patruno, Maria Anna De Lutiis, José Luis Quiles, Alfredo Grilli, Mario Felaco, Lorenza Speranza

**Affiliations:** 1 Department of Medicine and Science of Aging, University “G. D’Annunzio”, Chieti 66100, Italy; alessio.ferrone@yahoo.it (A.F.); danielagatta@unich.it (D.M.P.G); antonia.patruno@unich.it (A.P.); maria.delutiis@unich.it (M.A.D.L); mfelaco@unich.it (M.F.); lorenza.speranza@unich.it (L.S.); 2 Department of Psychological, Health and Territorial Sciences, University “G. D’Annunzio”, Chieti 66100, Italy; mirkopesce@unich.it (M.P.); algrilli@unich.it (A.G.); 3 Department of Physiology, Institute of Nutrition and Food Technology “José Mataix”, Biomedical Research Centre, University of Granada, Granada 18071, Spain; jlquiles@ugr.es

**Keywords:** inflammation, nitric oxide, licochalcone C, adhesion molecule, cardiomyocytes, nuclear factor-κB, Akt

## Abstract

Polyphenols compounds are a group molecules present in many plants. They have antioxidant properties and can also be helpful in the management of sepsis. Licochalcone C (LicoC), a constituent of *Glycyrrhiza glabra*, has various biological and pharmacological properties. In saying this, the effect of LicoC on the inflammatory response that characterizes septic myocardial dysfunction is poorly understood. The aim of this study was to determine whether LicoC exhibits anti-inflammatory properties on H9c2 cells that are stimulated with lipopolysaccharide. Our results have shown that LicoC treatment represses nuclear factor-κB (NF-κB) translocation and several downstream molecules, such as inducible nitric oxide synthase (iNOS), intercellular adhesion molecule-1 (ICAM-1) and vascular cell adhesion molecule-1 (VCAM-1). Moreover, LicoC has upregulated the phosphatidylinositol 3-kinase (PI3K)/protein kinase B (Akt)/endothelial nitric oxide synthase (eNOS) signaling pathway. Finally, 2-(4-Morpholinyl)-8-phenyl-1(4*H*)-benzopyran-4-one hydrochloride (LY294002), a specific PI3K inhibitor, blocked the protective effects of LicoC. These findings indicate that LicoC plays a pivotal role in cardiac dysfunction in sepsis-induced inflammation.

## 1. Introduction

Sepsis is a final common pathway to death from infection. It is characterized by a systemic inflammatory disorder, which leads to multiorgan failure with immune dysfunction [1]. Septic cardiac dysfunction, caused by bacterial endotoxin lipopolysaccharide (LPS), clearly manifests severe sepsis, making it the predominant cause of death in patients with sepsis [2,3]. Existing studies have shown that the mitigation of cardiac dysfunction in sepsis could improve the outcomes of patients with sepsis. Considering this evidence, treatments for sepsis with anti-inflammatory drugs have demonstrated a decreased risk of cardiovascular complications [4,5]. Nuclear factor kappa B (NF-κB) is an important transcription factor, as it activates several inflammatory genes after immune-stimulation. Generally, in unstimulated cells, NF-κB is retained in the cytoplasm bound with the inhibitor of NF-κB protein (IκB). Exposure to LPS leads to the activation of the IκB kinase (IKK) complex, inducing phosphorylation and subsequent degradation of IκB. Therefore, NF-κB is liberated and is subsequently translocated into the nucleus where it binds to DNA-response elements [6,7]. In septic cardiac dysfunction, the activation of these signaling cascades has been implicated in the release of genes activated by NF-κB, such as inducible nitric oxide synthase (iNOS), intercellular adhesion molecule-1 (ICAM-1) and vascular cell adhesion molecule-1 (VCAM-1) [8]. These molecules are present in elevated quantities in patients with an inflammatory state, such as sepsis, and may have detrimental effects on cardiomyocytes, leading to sepsis-associated myocardial dysfunction [9]. In this context, the activity of iNOS leads to excessive NO production that causes decreased vascular tonus and consequently hypotension, which is a characteristic of this syndrome. Instead, ICAM-1 and VCAM-1 are localized in the cell membrane of both the cells covering the vascular endothelium and leukocytes and are involved in the migration into adjacent tissues to inflammatory cells. Several experimental data evidenced that the inhibition of iNOS could be an important therapeutic target to treat septic shock patients and that the adhesion molecules (ICAM-1 and VCAM-1) have been relate with the gravity of the sepsis syndrome [10,11,12]. The phosphatidylinositol 3-kinase (PI3K)-protein kinase B (Akt) pathway plays an important role in many biological responses, including inflammatory reactions, chemotaxis, cellular activation and apoptosis. It also regulates inflammatory genes. A number of studies have shown that sepsis reduces myocardial PI3K/Akt signaling pathway activation [13,14]. The major signaling molecule of PI3K is Akt, an upstream enzyme of endothelial nitric oxide synthase (eNOS), unlike iNOS, which is responsible for the production of physiological amounts of nitric oxide (NO). The PI3K-Akt pathway has different effects on NF-κB and in turn on the expression of inflammatory molecules. Recent studies have highlighted that in HepG2 cells and in human monocytic cells, the PI3K-Akt pathway increases nuclear translocation of p65 [15,16]. Studies have also shown that in fibroblasts, an overexpression of Akt increases NF-κB-dependent gene expression [17]. In contrast, in regards to macrophages, C6 glioma cells and rat primary astrocytes activation of PI3K-Akt represses LPS-induced iNOS [18]. Over recent years, an increasing number of studies have suggested that several dietary compounds produce a variety of phytochemical compounds, such as polyphenols and flavonoids. These compounds have ample biological activities and can reduce the risk of disease. They also have a therapeutic potential on the cardiovascular system and have antihypertensive and anti-atherosclerotic effects [19,20,21]. Chalcones are a group of plant-derived polyphenolic compounds belonging to the flavonoids family and are characterized by a variety of cytoprotective functions due to their antioxidant activity. [22,23]. Firstly, we extracted Licochalcone C (LicoC) from *Glycyrrhiza glabra*, which was shown to decrease the inflammatory response in a monocytic cell line. This showed a reduction in iNOS expression and activity and a restoration in the antioxidant network activity of superoxide dismutase, catalase and glutathione peroxidase [24]. Moreover, recently, it has been reported that the cardioprotective effect of LicoC in myocardial ischemia/reperfusion injury may lead to antioxidant, anti-inflammatory and anti-apoptotic activities [25]. However, little is known on the molecular mechanisms of LicoC in cardiac cells. In this study, we investigated in vitro effects of LicoC on H9c2 cells protection and to elucidate its underlying mechanisms.

## 2. Results

### 2.1. Licochalcone C (LicoC) Does Not Affect the Viability of and Alleviated Lipopolysaccharide (LPS)-Induced Cytotoxicity in H9c2 Cells

**Figure 1:**
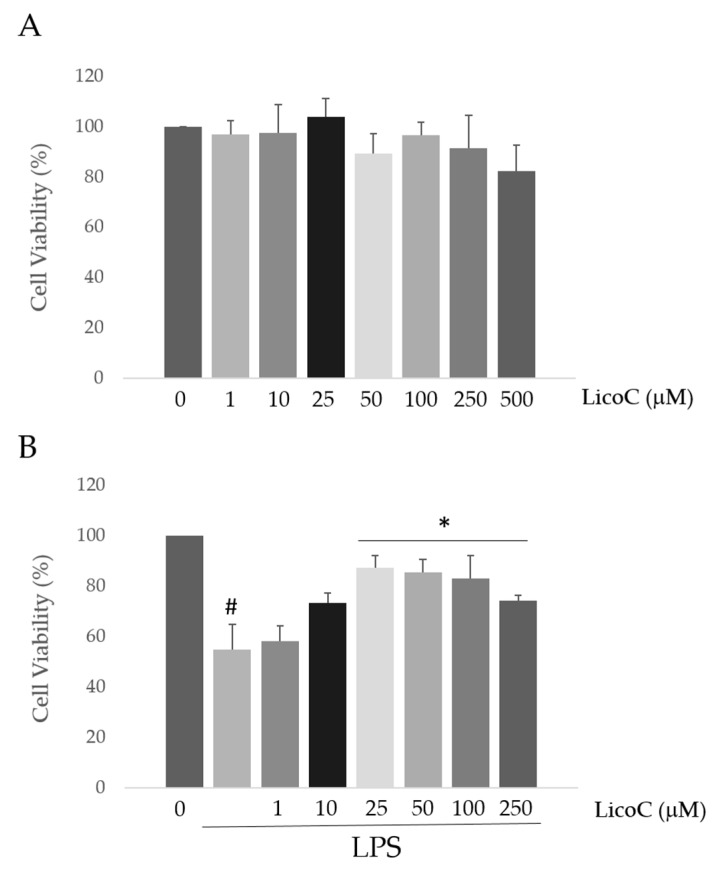

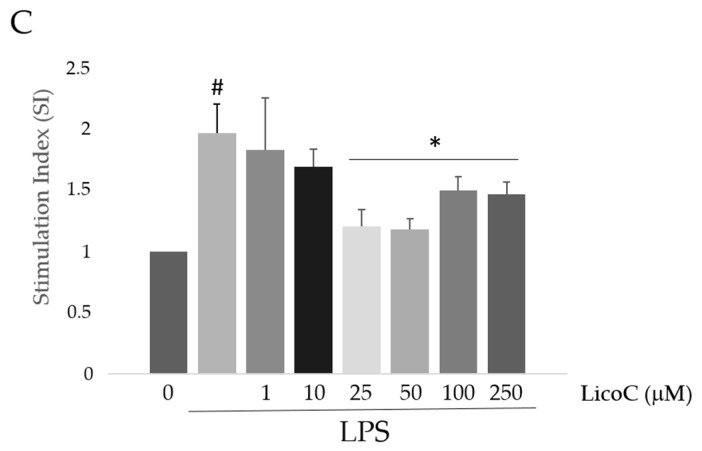
Effects of licochalcone C (LicoC) and/or Lipopolysaccharide (LPS) on H9c2 cells’ viability and toxicity. (**A**) Cell viability was measured by the 3-(4,5-dimethylthiazolyl-2)-2,5-diphenyltetrazolium bromide (MTT) assay, incubating H9c2 cells with increasing concentrations of LicoC (0–500 µM) for 24 h. Cell viability was not significantly affected by any tested LicoC concentrations; (**B**) LPS decreased the viability of H9c2 cells, but treatment with LicoC at concentrations greater than 10 µM restores cell viability; (**C**) Cells were treated with LicoC (1–250 µM) and LPS for 24 h. Reactive Oxygen Species (ROS) production was measured by the nitro blue-tetrazolium (NBT) assay. # *p* < 0.05 vs. control cells; ∗ *p* < 0.05 vs. LPS.

In this study, the effects of LicoC on cell viability in a model of a cardiomyocyte cell line, H9c2, were first investigated by the 3-(4,5-dimethylthiazolyl-2)-2,5-diphenyltetrazolium bromide (MTT) assay. When cells were treated with LicoC, ranging from 1–500 µM for 48 h, cell viability did not change significantly with respect to the control up to 250 µM (Figure 1A). Subsequently, we evaluated the effect of LicoC on LPS-induced inflammation. It was found that LPS treatment for 24 h caused about 55.04% ± 9.78% of cell death (Figure 1B). However, pretreatment with LicoC significantly protected cells from insults induced by LPS in a concentration-dependent manner. Statistically, a significant inhibition effect on the cytotoxicity of LicoC commenced at 25 µM. As LPS quickly stimulated reactive oxygen species (ROS) generation, to assess whether LicoC reduces LPS-induced ROS production in H9c2 cells, cells were co-treated with LicoC (1–250 µM) or/and LPS. Furthermore, the level of ROS reflected by the nitro blue-tetrazolium (NBT) assay was evaluated. As expected, the ROS level was much higher in the cardiomyocytes stimulated with LPS with respect to control cells, while as cells are incubated with LicoC, we observed a significant reduction of ROS production (Figure 1C). LicoC significantly reduced the LPS-induced ROS generation at concentrations greater than 10 µM, indicating that LicoC was able to reduce intracellular ROS accumulation after LPS stimulation. Thus, LicoC at 25 µM was more often used in the following experiments to test the role of this natural compound against LPS-induced effects.

### 2.2. Effect of LicoC on Nuclear Factor Kappa B (NF-κB)/Inducible Nitric Oxide Sinthase (iNOS)/Nitric Oxide (NO) Pathway

**Figure 2:**
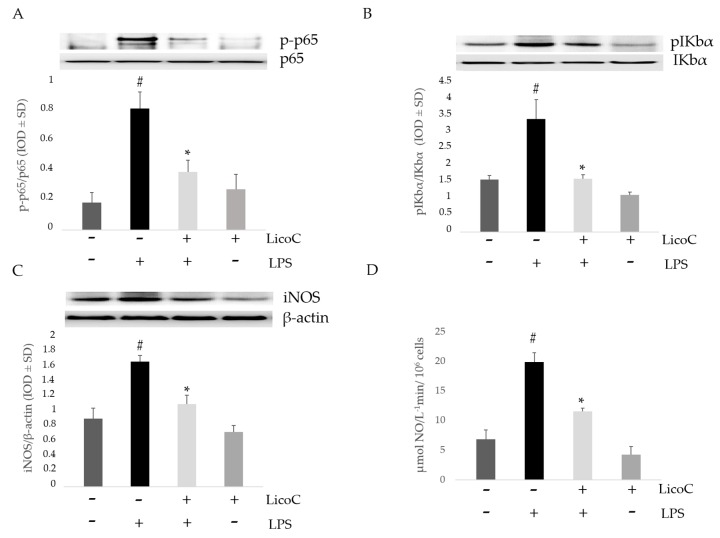
Effect of LicoC on NFκB/iNOS/NO signaling in LPS-stimulated H9c2 cells. Cells were pretreated with LicoC (25 µM) for 30 min and were then stimulated with LPS (10 µg/mL) for 24 h. After the indicated time, cells were harvested and lysed. Western blot analysis, in the absence (−) or presence (+) of LicoC or LPS, was used to investigate p-p65 NF-κB nuclear protein (**A**), as well as to detect the cellular expression of pIKBα (**B**) and iNOS (**C**). Equal loading was verified using an un-phosphorylated isoform and an anti-β-actin antibody. Densitometric analysis was carried out using Bio-Rad Quantity One Analysis software. Each column represents the mean ± SD of three independent experiments; (**D**) Effects of LicoC on NO levels in H9c2 cells stimulated by LPS. NO levels were quantified by the accumulation of nitrite in the cell culture medium and are expressed as µmol·L^−1^/10^6^ cells. Data are expressed as the mean ± SD of different experiments performed in triplicate; * *p* < 0.05 vs. LPS-treated cells; # *p* < 0.05 vs. control cells. Data were analyzed by one-way ANOVA followed by Bonferroni’s test.

The nuclear translocation of NF-κB p65 was assessed by Western blot analysis to verify the anti-inflammatory effects of LicoC on H9c2. As shown in Figure 2A, LPS producesa nuclear translocation of p65 in H9c2 cells. However, the pre-treatment with LicoC blocks the nuclear translocation of NF-κB p65 from the cytosol. Thus, we analyzed if the LicoC effect on NF-κB nuclear translocation was associated with the IκB phosphorylation. The activation of H9c2 cells with LPS leads to Iκ-Bα phosphorylation, allowing for the relocation of NF-κB to the nucleus. Pretreatment with our compound blocked Iκ-Bα phosphorylation, preventing p65 nuclear translocation induced by LPS (Figure 2B). We found that LicoC decreases LPS-induced inflammatory responses in H9c2 cells by blocking the activation of the transcription factor NF-κB. We then assessed the effect of LicoC on iNOS/NO signaling. Protein analysis demonstrated a higher expression of iNOS in activated cells compared to control cells, whilst LicoC-treated cells significantly decreased it (Figure 2C). This upregulation of iNOS expression is also associated with an increased enzyme activity, as seen in the analysis of culture medium levels of NO by using the Griess assay. Compared to the medium-control, LPS on its own significantly increased NO levels, whilst LicoC at a concentration of 25 µM markedly reduced the production of NO in LPS-activated cells, in comparison to control cells (Figure 2D).

### 2.3. LicoC Elevated PI3-K/Akt/eNOS Pathways

**Figure 3:**
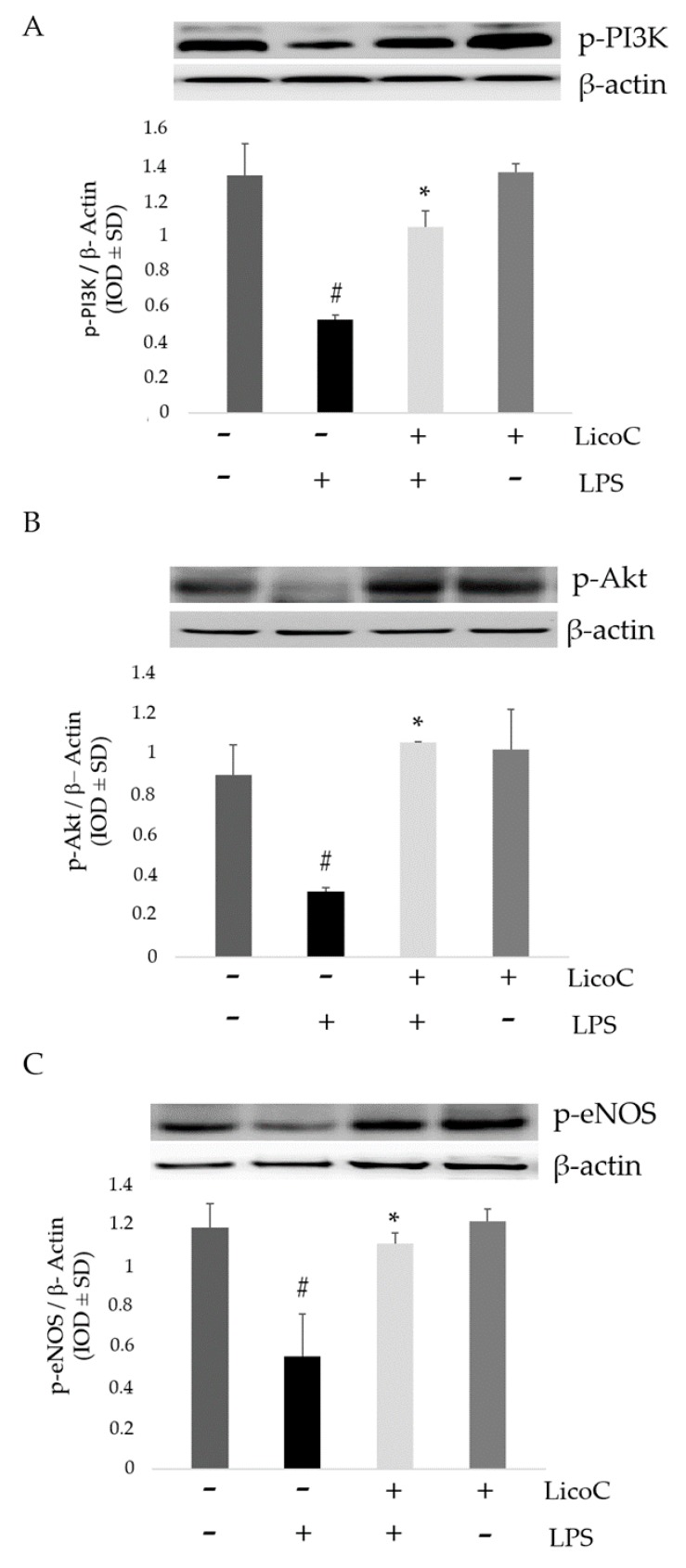
Effect of LicoC on PI3-K/Akt/eNOS pathways. H9c2 cells were pretreated with LicoC (25 µM) for 30 min and then stimulated with LPS (10 µg/mL). The figure shows representative Western blotting and a relative densitometry of p-PI3K (**A**), p-Akt (**B**) and p-eNOS (**C**) in whole cell extracts in presence (+) or absence (−) of LPS or LicoC. β-actin was used as the internal control. Values are expressed as the mean ± SD (*n* = 3). * *p* < 0.05 vs. LPS-treated cells; # *p* < 0.05 vs. control cells.

To strengthen our hypothesis about the protective mechanism of LicoC on cardiomyocyte cells, expressions of PI3-K/Akt signaling molecules were determined through Western blotting analysis. The analysis for phospho-specific and active isoforms of PI3-K and Akt was performed on homogenate cells subjected to pro-inflammatory stimulus and/or LicoC. As shown in Figure 3A,B, the densitometric analysis of phospho-PI3K and phospho-Akt demonstrated that in cells subjected to stress, there was a decrease in PI3K and Akt phosphorylation compared to control cells. It has been shown that phosphorylation of Akt is an important cellular signaling event for eNOS activation, resulting in the protection of cardiomyocytes [26]. Therefore, to further determine the underlying mechanism of the effects of LicoC on H9c2 cells, we tested the expression of eNOS using a specific antibody. Western blot analysis of eNOS protein demonstrated the reduction of a band in the cell activated with LPS (Figure 3C). When we treat the cells with LicoC, we saw both an increase in phosphorylation levels of eNOS and an increase in pAkt and pPI3K expressions. No changes in β-actin expression were evident between the control cells, nor the LPS-LicoC-incubated cells.

### 2.4. LicoC Inhibits Intercellular Adhesion Molecule 1 (ICAM-1) and Vascular Cell Adhesion Protein 1 (VCAM-1)

**Figure 4:**
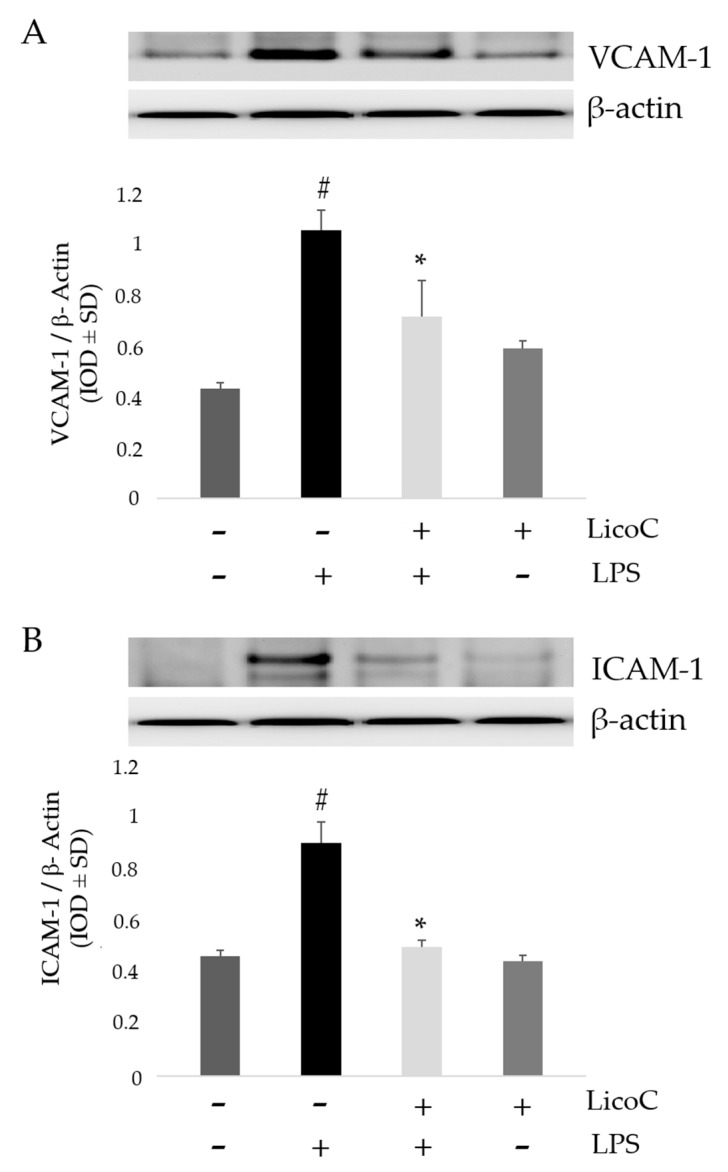
Effect of LicoC on adhesion molecules Intercellular Adhesion Molecule 1 (ICAM-1) and Vascular cell adhesion protein 1 (VCAM-1). VCAM-1 and ICAM-1 protein expression was reduced by treatment with LicoC upon LPS stimulation. H9c2 cells were pretreated for 30 min with LicoC (25 µM) and then stimulated with LPS (10 µg/mL). The figure shows the immunoblot and densitometric analysis of one experiment representing VCAM-1 (**A**) and ICAM-1 (**B**) expression on H9c2 cells in presence (+) or absence (−) of LPS or LicoC. β-actin was used as the internal control. Values are expressed as the mean ± SD (*n* = 3). # *p* < 0.05 vs. LPS-treated cells; * *p* < 0.05 vs. control cells.

To further understand the cardio-protective role of LicoC and to assess the potential signaling pathways contributing to the properties of this natural compound, we analyzed the expression of adhesion molecules VCAM-1 and ICAM-1 in H9c2 cells after LPS stimulation. Figure 4 shows that LPS stimulation induces a significant upregulation of the expression of VCAM-1 and ICAM-1 compared to control cells (*p* < 0.01). However, treatment with LicoC leads to a marked reduction of VCAM-1 and ICAM-1 levels by 20.4% and 34.0%, respectively, in LPS-stimulated cells when compared to activated cells that were not treated with LicoC (*p* < 0.01). These data suggest that LicoC acts on cardiomyocytes to produce an anti-inflammatory effect.

### 2.5. Involvement of PI3K on the Protective Effect of LicoC on LPS-Induced H9c2 Inflammation

**Figure 5:**
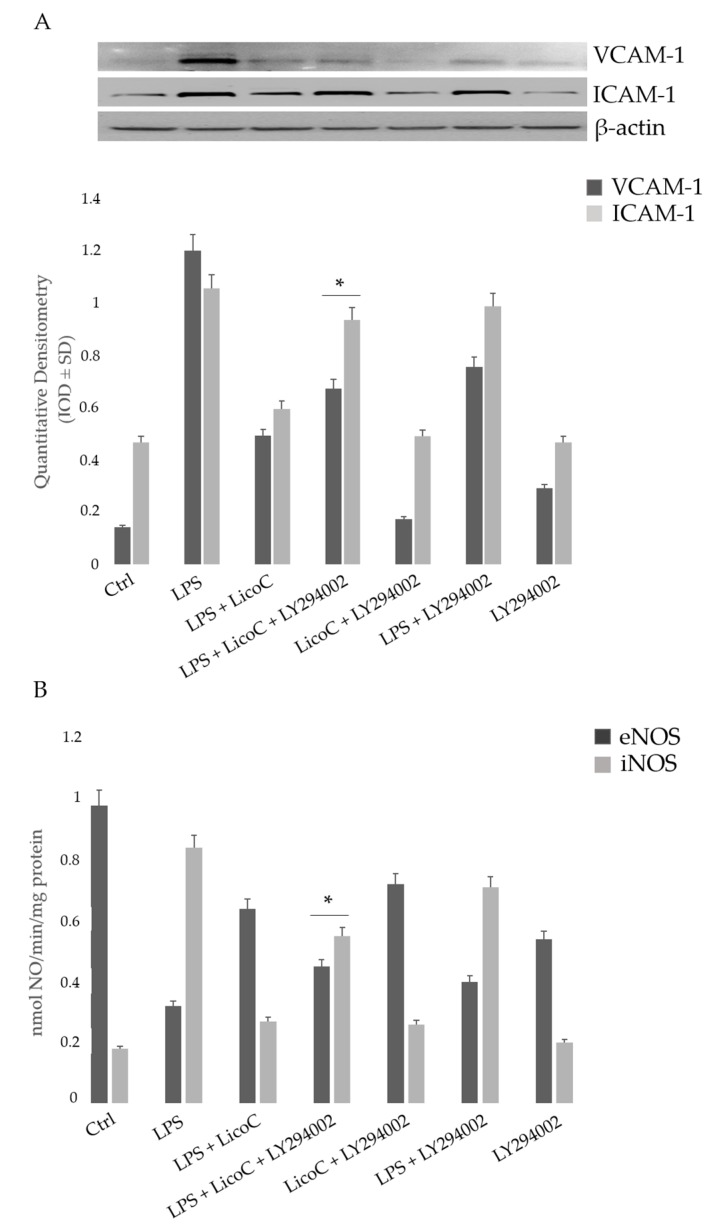
Effect of LicoC on adhesion molecules and on NOS activity in H9c2 cells treated with LPS, LicoC, 2-(4-Morpholinyl)-8-phenyl-1(4*H*)-benzopyran-4-one hydrochloride (LY294002) or all (LPS, LicoC, LY294002). Cells were pretreated with LicoC (25 µM) and LY294002 (10 µM) for 30 and 60 min respectively before stimulation with LPS (10 µg/mL) for 24 h. VCAM-1 and ICAM-1 protein expression was normalized to β actin (**A**); eNOS and iNOS activity (**B**) values are expressed as the means ± SD of three experiments. * *p* < 0.05 vs. LPS + LicoC.

To investigate the role of PI3K/Akt signaling activation on the cardio-protective effect of LicoC against LPS-induced inflammation, we pre-treated cells with a selective PI3K inhibitor LY294002 (2-(4-Morpholinyl)-8-phenyl-1(4*H*)-benzopyran-4-one hydrochloride). As shown in Figure 5A, LY294002 blocks the preserved activation of the eNOS induced by LicoC in LPS stimulated cells. We then studied whether the inhibition of PI3K/Akt/eNOS signaling with LY294002 modified the iNOS activity and the protein expression of adhesion molecules ICAM-1 and VCAM-1. Our experimental analysis showed that the LicoC-induced reduction of ICAM-1 and VCAM-1 proteins expression and iNOS activity was blocked by the treatment of cells with the PI3K-specific inhibitor LY294002, demonstrating that they need PI3-K/Akt activation. LY294002 eliminated the protective effect of LicoC on the attenuation of cellular homeostasis alteration in LPS-induced sepsis. These data highlight that LicoC exerts its effect by activating the PI3K/Akt signaling pathway.

## 3. Discussion

Sepsis is a serious clinical problem related to a poor prognosis characterized by severe systemic inflammatory response to infection, possibly leading to cardiac dysfunction. This study aimed to investigate the mechanisms responsible for the putative protective effect of LicoC on cultured H9c2 cardiomyocytes. It has been shown that this cell line exhibits a marked inflammatory response to LPS stimulation. Thus, the H9c2 cell line is a sufficient model to study the therapeutic potential of molecules for the treatment of cardiac dysfunction induced by sepsis [27]. Firstly, we found that in cardiomyocytes, LicoC does not reduce viability, but does exhibit significant attenuation of LPS-induced toxicity in the H9c2 cell line (Figure 1A,B). Oxidative stress, a condition caused by excessive production of free radicals, plays an important role in the progression of an inflammatory condition [28,29]. LPS, released from the surface of the cell membrane of Gram-negative bacteria, triggers an inflammatory response and causes severe sepsis. The cellular response to LPS includes the production of ROS and other mediators, such as NO and pro-inflammatory cytokines [30,31]. Therefore, we studied the possibility of LicoC in decreasing the cellular accumulation of ROS LPS-induced. As shown in Figure 1C, LicoC has a significant decrease in ROS production on H9c2 cells activated with LPS. To demonstrate how LicoC plays a role in the inflammation process, we analyzed its effects on H9c2 cells. H9c2 cells exposed to LPS show an increased activation of NF-κB. The transcription factor NF-κB plays an important role in LPS-induced transcriptional regulation, controlling various target inflammatory genes, such as acute phase proteins, cell adhesion molecules, cytokines and chemokines and stress-responsive genes [32,33]. As shown in Figure 2A,B, treatment with LicoC, inhibited Iκ-Bα phosphorylation, prevents the nuclear translocation of p65 and increases the expression and activity of iNOS induced by LPS. These data suggest that the biological effect exerted by LicoC towards LPS-induced cardiac inflammation was associated with the inhibition of nuclear translocation of NF-κB. As many studies have demonstrated that in some cell lines, PI3K/Akt activation negatively regulates the NF-κB activation pathway, limiting pro-inflammatory responses, improving cardiac function in septic mice [34,35,36], we have examined whether the inhibition of NF-κB activation by LicoC is mediated via the PI3K/Akt pathway. PI3K/Akt signaling is an important pathway involved in controlling cardiomyocytes function and survival. It has been previously shown that one of the downstream effectors of Akt in the PI3K/Akt pathway is eNOS, which after phosphorylation, leads to the production of NO [37,38]. Endothelial-derived NO acts as a protective agent in a variety of diseases. It plays a pivotal role in the cardiovascular system as a key secondary messenger in the signaling pathway through the regulation of physiological activities, such as regional vascular tone; the maintenance of vascular integrity and leukocyte adhesion to the endothelium, as well as angiogenesis [39]. Our results show that the biological effects of LicoC are linked to the PI3K/Akt/eNOS pathway by promoting the phosphorylation of these proteins (Figure 3). Constitutively expressed eNOS has favorable effects on cellular function, while iNOS has adverse effects on cells mostly under conditions of stress, including sepsis. In our previous study, we observed that, in H9c2 cells, LPS markedly decreased iNOS expression and increased eNOS expression, suggesting the presence of an imbalance between iNOS and eNOS after LPS stimulation [3]. During sepsis, this imbalance could contribute to alteration of microvascular tone and integrity, as well as the activation of cell adhesion molecules, such as VCAM-1 and ICAM-1. Interestingly, LicoC pre-treatment significantly abrogated the downregulation of eNOS and the upregulation of iNOS (Figure 3B,C). This suggests that the cardioprotective effect of LicoC against LPS stimulation could be related to the maintenance of the balance between the constitutive and inducible isoforms of NOS during inflammation. Findings from previous studies have shown that in addition to iNOS, VCAM-1 and ICAM-1 are also inflammatory molecules primarily activated by NF-κB [40]. Elevated expression of adhesion molecules, such as VCAM-1 and ICAM-1, appears to be important in the controlling of lymphocyte migration from the vasculature into the myocardium, which in turn leads to the reduction of left ventricular contractility after the triggering of an inflammatory process [41]. In accordance with this experimental evidence, we observed that in LPS-activated H9c2 cells, the expression levels of adhesion molecules, VCAM-1 and ICAM-1, have significantly increased. We can see that the upregulation of ICAM-1 and VCAM-1 by LPS has been effectively abrogated by LicoC treatment (Figure 4). This shows that LicoC reduces inflammation in LPS-activated cells. Finally, inhibition of PI3K/Akt signaling by LY294002 repressed the LicoC-induced cellular protection during endotoxemia, which resulted in the increased activity, as well as the expression of molecules related to inflammation, such as iNOS, VCAM-1 and ICAM-1, regulated by transcription factor NF-κB (Figure 5). Moreover, we noticed a reduction in the activity of a key molecular implicated in the maintenance of endothelial function, eNOS protein. These results suggest a direct involvement of the PI3K/Akt pathway in LicoC-induced NF-κB deactivation.

## 4. Materials and Methods

### 4.1. Cell Culture

H9c2 cells were purchased from the American Type Culture Collection (ATCC, Manassas, VA, USA). Cells were cultured in a 5% CO_2_ atmosphere in DMEM with 10% Fetal Bovine Serum (FBS), 100 ng/mL streptomycin, 100 U/mL penicillin and 2 mM l-glutamine. Then, 2 × 10^5^ cells were seeded onto culture plates and cultured in medium with Lipopolysaccharide (LPS) (10 µg/mL) for 24 h, with or without LicoC and/or LY294002, 10 µM, Sigma-Aldrich, St. Louis, MO, USA, Catalog Number L9908). LicoC and PI3K inhibitor LY294002 were added to the culture medium 30 and 60 min, respectively, before the LPS treatment. After incubation time, cells were collected for analysis of cellular viability, gene and protein expression. Culture medium was retained to estimate the NO level.

### 4.2. 3-(4,5-Dimethylthiazolyl-2)-2,5-diphenyltetrazolium Bromide (MTT) Assay for Cell Viability and Cytotoxicity

To assess the cell viability and toxicity of LicoC, the MTT assay was used, which was performed as described above [42]. Briefly, the cells were seeded on 96-well plates (8 × 10^3^ cells/well), and the reagent MTT at a concentration of 0.5 mg/mL (20 µL) and medium (200 µL) were added to each well. The plates were incubated at 37 °C (4 h). Then, the solution (220 µL) was removed, and DMSO (150 µL) was added. The reading of the amount of reduced MTT was measured on an ELISA reader (Bio-Rad, Hercules, CA, USA) at 570 nm. The following equation was used to calculate the percentage of cell viability:

% = (Absorbance of treated cells)/(Absorbance of control cells) × 100

### 4.3. Nitro Blue-Tetrazolium Assay

Superoxide anion production was monitored using the nitro blue-tetrazolium (NBT) assay. As described previously [43], to each well were added: 100 µL PBS (pH 7.8, 50 mM), 5 µL catalase, 25 µL NBT (5.6 × 10^−9^ M), 50 µL xanthine (0.1 mM), 50 µL xanthine oxidase (0.1 mM) and LicoC (1–250 µM). Following the addition of NBT, the plates were incubated at RT for 1 h, and the amount of NBT formazan was quantified at 560 nm.

### 4.4. Western Blot Analysis

For Western blot analysis, the cells were harvested with RIPA lysis buffer as described previously [44]. Nuclear extracts were prepared as described above [45]. The Bradford Method was used for protein quantification. Western Blot analysis was performed as described previously [46] incubating the membrane with antibodies against IKB, pIκB, p65-NFκB, p-p65-NFκB, VCAM-1 and ICAM-1 (Santa Cruz Biotechnology, Santa Cruz, CA, USA) (dilution 1:500). β-actin was used as a protein loading control.

### 4.5. Griess Assay

The assay was performed as described above [3]. Briefly, the cells were seeded, and nitrite was measured in culture medium supernatants as an indicator of the NO levels. To assess nitrite concentration, sodium nitrite was used as a standard. Aliquots of the culture supernatant were mixed with an identical volume of the Griess reagent, and absorbance was detected spectrophotometrically at 540 nm.

### 4.6. Nitric Oxide Sinthase (NOS) Activity

The oxyhemoglobin assay was performed to detect nitric oxide formation from NOS as described above [47]. The reaction mixture for the evaluation of NOS activity was constituted by CaCl_2_ (1.6 mM), l-arginine (10 µM), calmodulin (11.6 mg/mL), tetrahydrobiopterin (6.5 µM), dihydronicotinamide-adenine dinucleotide phosphate (NADPH, 100 µM) and oxyhemoglobin (3 mM) in 4-(2-hydroxyethyl)-1-piperazineethanesulfonic acid (HEPES, 100 mM) (pH 7.5) in a final volume of 1 mL. iNOS activity was performed in calcium-free conditions. The quantization of methemoglobin as the product of the reaction between nitric oxide and oxyhemoglobin was detected at 576 nm (*e* = 12.000 M^−1^·cm^−1^) using a Perkin-Elmer LamdaBIO UV-Vis spectrophotometer (Perkin-Elmer, Milano, Italy).

### 4.7. Statistical Analysis

All results are reported as the means ± SD. To show the difference between samples, considered as statistically significant.

## 5. Conclusions

Our study demonstrates a new mechanism of action through which LicoC positively modulates the functional recovery and integrity of endothelial function. This occurs through the controlling of both NO concentration and expression of adhesion molecules. Therefore, LicoC may be used as an adjuvant treatment in order to reduce cardiomyocyte inflammation.

Provided that the cardioprotective effect is mediated by LicoC in vitro through the negative modulation of closely-related molecules to inflammatory etiopathogenesis, it would be desirable to undertake a further study of an evaluation of the same pathways (PI3K/Akt/eNOS) in vivo.

## Abbreviations

LPS	lipopolysaccharide
iNOS	inducible nitric oxide synthase
ICAM-1	intercellular adhesion molecule-1
eNOS	endothelial nitric oxide synthase
ROS	reactive oxygen species
PI3K	phosphoinositide 3-kinase
VCAM-1	vascular cell adhesion molecule-1
